# Dietary gamma-linolenic acid supports arachidonic acid accretion and associated Δ-5 desaturase activity in feline uterine but not ovarian tissues*

**DOI:** 10.1017/jns.2014.41

**Published:** 2014-10-13

**Authors:** Amy J. Chamberlin, John E. Bauer

**Affiliations:** 1 Department of Small Animal Clinical Sciences, Companion Animal Nutrition Research Laboratory, College of Veterinary Medicine and Biomedical Sciences, Texas A&M University, College Station, TX 77843–4474, USA; 2 Intercollegiate Faculty of Nutrition, Texas A&M University, College Station, TX 77843–4474, USA

**Keywords:** Feline nutrition, Reproduction, Arachidonate, Δ-5 Desaturase, ARA, arachidonic acid, DGLA, di-homo γ-linolenic acid, GL, high γ-linolenic acid, GLA, γ-linolenic acid, HL, high linolenic acid, LA, linoleic acid, LL, low linolenic acid, PL, phospholipid

## Abstract

Arachidonic acid (ARA) is essential in felines because conversion of dietary linoleic acid (LA) to ARA is rate-limited by low Δ6-desaturase. Dietary γ-linolenic acid (GLA) may serve as an ARA precursor by-passing this initial rate-limiting step. This possibility was investigated using twenty-six adult female domestic shorthair cats divided into three groups and fed on complete and balanced diets containing high GLA (GL), high LA (HL) or low LA (LL, control) diets, for 300 d prior to ovariohysterectomy. Plasma was obtained 1–2 d before surgery and uterine, ovarian and associated adipose tissues were reserved for lipid analysis. Fatty acid profiles of the plasma phospholipid (PL) fractions and adipose lipids were performed. In the GL group, plasma and uterine tissue PL were significantly enriched in GLA, di-homo GLA (DGLA) and ARA compared with control. However, ovarian and adipose tissue PL were only enriched in DGLA. Enrichment of uterine tissues with DGLA and ARA probably supplies the essential eicosanoid precursors for reproduction when GLA is fed consistently with an active Δ5-desaturase in uterus. By contrast, this enzyme appears less active in ovary because ARA was not higher compared with control. Earlier reports concluded that ARA was not necessary for fertilisation (an ovarian function), but required for successful pregnancy and reproduction (a uterine function). Adipose tissue DGLA may be a reservoir for ARA synthesis by other tissues upon mobilisation. Dietary GLA may meet feline ARA requirements in the absence of an animal-based preformed source of ARA.

Feline lipid metabolism is unique among animal species. Most mammalian species convert linoleic acid (LA, 18 : 2*n*-6) to arachidonic acid (ARA, 20 : 4*n*-6) via a cascade of enzymatic steps, including ∆6-desaturation, chain elongation and ∆5-desaturation^(^^1^^)^. Nevertheless, earlier studies determined that cats seemed to lack sufficient Δ6-desaturase activity and that ARA was a necessary dietary essential for feline species^(^^2^^–^^4^^)^. Later on, using the stable isotopes, detectible amounts of both Δ6- and Δ5-desaturase products in feline liver were found^(^^5^^)^, but it remained to be determined whether ARA synthesis was sufficient for all life stages. Where reproduction is concerned, cats fed an ARA-free diet containing hydrogenated coconut oil found that males did not require ARA for growth or reproduction^(^^6^^)^. Females, fed similar diet, were also able to grow, enter oestrus and conceive. However, after conception they produced only a few viable kittens^(^^7^^)^ It was concluded that ARA and possibly other PUFA are needed to complete normal full-term pregnancies.

A recent study of plasma phospholipid (PL) fatty acids from our laboratory found evidence for Δ5-desaturation in feline species fed dietary GLA^(^^8^^)^. However, in view of the earlier findings regarding feline reproduction, it was subsequently hypothesised that feeding cats a GLA-enriched diet by-passes the Δ6-desaturase in uterine and ovarian tissues thereby providing precursors for ARA synthesis in these tissues.

## Experimental methods

The present investigation was approved by the Texas A&M University Animal Care and Use Committee. Twenty-six, sexually intact female cats ranging between 1½ and 2 years of age were used in the study. They had been carefully fed *ad libitum* over a 300 d period on the diets employed in the present study according to their metabolic body weights using the equation, Daily Metabolisable Energy = 418·4 kJ (100 kcal) ME × W_kg_^0·67^ per d with water. Daily consumption records and weekly body weights were recorded. Body condition scores using a 1 to 9 scale, where 5 is ideal, were evaluated exclusively by one of the investigators and used to adjust amounts fed. In this way, a range of body condition score values between 5 and 6 was achieved with a median value of 5 over the entire feeding period. Body weights of the cats ranged from 2·4 to 3·9 kg with a median value of 3·29. All cats were housed individually at the Laboratory Animal Research Resources facility, Texas A&M University, according to the American Physiological Society Guidelines for Animal Research and guidelines set forth by Texas A&M University Care and Use Committee. Each cage for cats was 2·5 m long, 2·0 m high and 1·2 m wide. Prior to the investigation and at intervals throughout the study physical examinations, complete blood counts, serum biochemistry profiles and T_3_, thyroid-stimulating hormone assays were performed on all cats to verify their normal clinical status.

The cats were cared for by the Laboratory Animal Research and Resources staff and resident veterinarian. Members of the Companion Animal Nutrition Laboratory monitored and fed the cats daily. These protocols were approved by Texas A&M University Animal Care and Use Committee. Previously, the animals had been randomly assigned to one of three diet groups for a separate project lasting 56 d. They were then continued on their respective diets thereafter for 300 d for the present study^(^^8^^)^. Twenty-nine cats had been originally randomised using a computer spreadsheet randomisation function (Microsoft^®^ Excel 2008), but three were excluded prior to 300 d for non-dietary related concerns. The earlier study revealed suitable statistical power for plasma PL fatty acid profile differences using 8–10 cats per group when fed carefully on controlled diets^(^^8^^)^. In addition, plasma PL are generally considered to reflect tissue levels under steady-state conditions and this condition would be likely after 300 d of feeding. Thus, the sample size of the present study was considered to be adequately powered to detect tissue differences. The diets in the present study were complete and balanced containing either high γ-linolenic acid (GL, *n* 10), high linoleic acid (HL, *n* 7) or low linoleic acid (LL, *n* 9, control), for a total of 300 d prior to ovariohysterectomy. The HL diet used LA-rich safflower oil and the LL diet contained coconut oil in place of safflower oil to reduce LA to low, but adequate, levels. The GL diet was enriched with high GLA borage oil containing 70 % GLA plus an adequate amount of LA. The three diets were comparable in all respects except for omega-6 fatty acid type, while the omega-3 contents of all diets were similar and kept to a minimum by design (Table 1). The nutrient composition and major PUFA contents of the diets were determined (Nestlé Purina Laboratories) and found to be within the expected analytical variance for the formulated targets of 350 (sd 15) g/kg DM for protein, 190 (sd 27) g/kg DM for fat, 75 (sd 11) g/kg DM for ash and 20 (sd 5) g/kg/DM for fibre (Table 1).

**Table 1.** Experimental diet nutrient and PUFA profiles (g/kg DM)

Diet component	Diet		
	GL	HL	LL (control)
	g/kg DM	g/kg DM	g/kg DM
Crude protein	346	355	349
Fat	185	199	182
Ash	71	77	70
Fibre	21	18	20
PUFA			
*n*-6			
LA (18 : 2*n*-6)	20·0	65·8	18·7
GLA (18 : 3*n*-6)	3·8	0·2	<0·1
ARA (20 : 4*n*-6)	0·30	0·31	0·31
*n*-3			
ALA (18 : 3*n*-3)	1·2	1·8	1·3
Other *n*-3	<0·1	<0·1	<0·1
	%	%	%
Moisture	7·7	8·7	8·4
	kJ/kg	kJ/kg	kJ/kg
Energy	18480·7	18058·1	18062·3

Cats were spayed after 300 d of feeding using the standard surgical techniques. Plasma samples were collected into tubes containing EDTA as anticoagulant 1–2 d before surgery, and ovaries, uterus and adjacent adipose tissues were harvested, trimmed of excess connective tissues and frozen at −80°C for the subsequent analysis. Total lipids were extracted from tissue homogenates prepared in phosphate-buffered saline (pH 7·4) and plasma. PL were fractionated from plasma, ovarian and uterine tissues and total the lipids were extracted from adipose tissues. Fatty acid methyl esters of the extracts were prepared and analysed via capillary GC^(^^9^^)^.

All data are presented as mol/100 mol of fatty acids and sd and evaluated using SPSS 16·0 for Windows (SPSS Inc, Chicago, IL). To determine whether the data were normally distributed, the Shapiro–Wilks test was performed. One-way ANOVA was utilised when data appeared normally distributed for diet comparisons. Where significant diet group differences were found, Tukey multiple comparison tests were performed (*P* < 0·5). Non-normally distributed data were analysed using the Kruskal–Wallis test. All results were considered statistically significant if the two-tailed *P* value was <0·05.

## Results

Significant increases in plasma PL di-homo GLA (DGLA) and ARA were observed with the GL diet (*P* = 0·009 and 0·007, respectively), while the HL and LL diets resulted in lower, and similar, amounts of DGLA (Table 2). It is of interest that plasma PL ARA was significantly lowest and plasma PL LA was significantly highest with the HL diet, whereas the GL diet was lowest and the LL (control) diet intermediate in value for each of these fatty acids (Table 2). It appeared that a relative enrichment of the plasma PL fraction with LA had occurred at the expense of ARA when the HL diet was fed. Similar to plasma, uterine tissue homogenate PL had statistically significantly higher amounts of DGLA and ARA with the GL diet (*P* < 0·001 in both cases), but GLA was not different compared with the other diet groups. Uterine LA was again significantly higher with the HL diet compared with the GL and LL (control) diets (*P* = 0·005). In this case, unlike plasma, uterine ARA of cats fed HL and LL diets were similar.

**Table 2.** Major PUFA of feline tissues when diets after 300 d of feeding (mol/100 mol)

Tissue	GL diet		HL diet		LL diet (control)	
	Mean or (median)	SD or (range)	Mean or (median)	SD or (range)	Mean or (median)	SD or (range)
LA (18 : 2*n*-6)						
Plasma	21·5^a^	2·8	35·7^b^	1·9	28·5^c^	1·9
Uterus	8·6^a^	0·3	16·8^b^	0·8	13·9^c^	1·1
Ovary	7·7	7·7	9·6	4·6	8·7	2·6
Adipose	16·2^a^	0·7	32·3^b^	0·9	15·2^a^	0·5
GLA (18 : 3*n*-6)						
Plasma	0·6^a^	0·3	0·2^b^	0·1	0·3^b^	0·1
Uterus	(0·0)	(0·0–0·2)	(0·0)	(0·0–0·1)	(0·0)	(0·0–0·7)
Ovary	0·4	0·6	0·4	0·8	0·3	0·6
Adipose	(0·6)^a^	(0·04–0·7)	(0·0)^b^	(0·0–0·1)	(0·0)^b^	(0·0–0·6)
DGLA (20 : 3*n*-6)						
Plasma	10·6^a^	4·2	0·8^b^	0·3	1·9^b^	0·2
Uterus	4·0^a^	0·9	1·2^b^	0·5	1·5^b^	0·5
Ovary	4·0^a^	1·1	1·9^b^	1·4	2·0^b^	1·1
Adipose	0·6^a^	0·2	0·06^b^	0·08	0·12^b^	0·08
ARA (20 : 4 *n*-6)						
Plasma	5·3^a^	0·9	2·2^b^	0·6	4·1^c^	0·4
Uterus	15·8^a^	0·7	10·3^b^	0·8	12·1^b^	0·7
Ovary	12·6	3·0	10·0	2·3	11·5	3·3
Adipose	0·20^a^	0·05	0·07^b^	0·03	0·15^a,b^	0·3

Values are mean and sd for parametric data, whereas those in parentheses are median and range of values for non-parametric data. GL diet (*n* 10), HL diet (*n* 7) and LL diet (*n* 9); superscript letters not in common in a row indicate statistically significant differences among diet groups; *P*<0·05.

A noteworthy finding from the present study was that ovarian tissue PL accumulated DGLA (*P* = 0·004), but no additional ARA with the GL diet compared with the other two diets. No other differences among the PUFA were observed in this ovarian tissue fraction. Adipose tissue lipid extracts revealed small, yet statistically significant enrichment of GLA and DGLA with the GL diet (*P* < 0·001 in both cases) with a marked accumulation of LA again observed with the HL diet (*P* < 0·03) (Table 2).

## Discussion

The accumulation of ARA, its DGLA precursor, and corresponding decrease of LA in plasma and uterine homogenate PL fractions of cats fed the GLA-enriched diet *v*. the other two diets was not unexpected. These findings confirm the results from our earlier study of plasma PL of cats fed these same three diets during a 56-d period^(^^8^^)^ and extend them to include fatty acid compositions of feline reproductive tissues and evidence for active Δ5-desaturation in uterine tissue. The uterine accretion of ARA with the GL diet would be expected to provide the substrate for PG synthesis in support of the successful full-term pregnancy^(^^10^^)^. Thus, a diet containing GLA from vegetable sources may be possible to meet feline ARA requirements for reproduction in the absence of a pre-formed animal-based ARA source.

By contrast, the lack of additional ARA accumulation in ovarian homogenate PL with dietary GLA was somewhat unexpected although DGLA was observed. This result suggests a low or absent of Δ5-desaturation in the ovarian tissue. Thus, in order for this tissue to become enriched with ARA it is likely to be the result of circulatory transport of ARA that has been either synthesised in other tissues or from direct dietary sources. This finding is consistent with the earlier reports by MacDonald *et al.* and by Morris who noted that dietary ARA did not seem to be needed for fertilisation to occur (an ovarian function), but necessary for successful pregnancy and viable kittens (a uterine function)^(^^6^^,^^7^^)^. Also, modest but statistically significant higher amounts of adipose GLA and DGLA when GLA is fed may provide a reservoir for tissues that possess active Δ5-desaturation allowing subsequent ARA synthesis.

Finally, it is noteworthy that higher amounts of LA were observed in plasma and uterine PL at the expense of ARA with high dietary LA (HL diet). This phenomenon was presumably due to competition between these two PUFA for incorporation into the PL fractions. Although it is unknown what level of ARA enrichment of tissue PL may be needed for optimal eicosanoid production in cats overall, the possibility exists that some threshold amount of ARA may be needed for optimal cell function. If so, the amount of LA used in the HL diet of the present study may help define a safe upper limit for dietary LA concentration in cats because high dietary amounts may effectively limit incorporation of ARA in the PL fractions.
